# Probabilistic Teleportation of Arbitrary Two-Qubit Quantum State via Non-Symmetric Quantum Channel

**DOI:** 10.3390/e20040238

**Published:** 2018-03-29

**Authors:** Kan Wang, Xu-Tao Yu, Xiao-Fei Cai, Zai-Chen Zhang

**Affiliations:** 1 National Mobile Communications Research Laboratory, Southeast University, Nanjing 210096, China; 2 State Key Lab. of Millimeter Waves, Southeast University, Nanjing 210096, China

**Keywords:** quantum teleportation, entanglement, quantum channel, quantum communication

## Abstract

Quantum teleportation has significant meaning in quantum information. In particular, entangled states can also be used for perfectly teleporting the quantum state with some probability. This is more practical and efficient in practice. In this paper, we propose schemes to use non-symmetric quantum channel combinations for probabilistic teleportation of an arbitrary two-qubit quantum state from sender to receiver. The non-symmetric quantum channel is composed of a two-qubit partially entangled state and a three-qubit partially entangled state, where partially entangled Greenberger–Horne–Zeilinger (GHZ) state and W state are considered, respectively. All schemes are presented in detail and the unitary operations required are given in concise formulas. Methods are provided for reducing classical communication cost and combining operations to simplify the manipulation. Moreover, our schemes are flexible and applicable in different situations.

## 1. Introduction

Quantum teleportation, firstly proposed by Bennett et al. [1], is a feasible technique for moving quantum states via pre-established quantum channel amongst distant network nodes with the help of classical information. It is at the heart of many quantum information protocols and also represents a fundamental ingredient to the development of many quantum technologies, including quantum network [2,3], quantum secure communication [4,5], measurement-based quantum computing [6,7], and quantum repeater [8,9,10], etc. Due to its potential applications in the realm of quantum communication [11], a growing amount of theoretical and experimental progress [12,13,14,15,16] has been made in this domain.

As the transmission channel of teleportation, quantum entanglement [17] is fragile resource. The requirement of a maximally entangled quantum channel connecting nodes is very difficult to achieve or maintain in practice since the inevitable presence of noise reduces the entanglement of the quantum state shared between them. In practical implementations of the teleportation protocol, one can either adopt entanglement purification and distillation techniques to purify the states or use the partially entangled state to teleport quantum state perfectly with some probability. Probabilistic teleportation was introduced by Li et al. [18], following which several resources have used different types of entanglement. These are obtainable in [19,20,21,22,23,24].

Agrawal et al. [19] utilized a partially entangled state as a shared resource to teleport an unknown two-qubit state. Dai et al. [20,21] presented two protocols for probabilistically teleporting an arbitrary two-qubit state via two partially entangled W states and by the combination of a partially entangled GHZ state and an entangled W state, respectively. Probabilistic teleportation of an arbitrary two-qubit entangled state can also be obtained via a dimensional four-qubit partially entangled cluster state by Xia et al. [22]. Liu et al. [23] proposed a teleportation protocol of an unknown two-qubit state probabilistically with partial information. Recently, Choudhury et al. [24] proposed protocol for probabilistic teleportation using POVM and projective measurement. Here, we focus on probabilistic teleportation schemes for an arbitrary two-qubit quantum state.

The existing works mostly use a symmetric quantum channel, i.e., two entangled states of two qubits or two entangled states of three qubits, to teleport two-qubit quantum state. However, the entangled states shared among quantum nodes in network would not be guaranteed to be of the same type. Different types of entanglements would be utilized as quantum channels. In this paper, we study the probabilistic teleportation using *non-symmetric quantum channel* for transmitting arbitrary two-qubit quantum state. The non-symmetric quantum channel consists of a two-qubit entangled state and a three-qubit entangled state. Schemes using different quantum channel combinations are proposed that could be seen as supplementary to the protocol family of teleporting two-qubit state. Methods are provided for reducing the classical communication cost and combining the separate unitary operations to simplify the whole process. Furthermore, in many existing protocols, the intermediate states and the unitary operations applied are shown in the form of complex tables. One of the unique features in this paper is that all unitary operations applied by the receiver and intermediate states in the process are summarized in concise formulas. With these formulas, the operations and intermediate states can be obtained through calculation rather than searching through complex tables.

The rest of this paper is organized as follows: Section 2 provides system model and quantum channels we considered in this paper. Section 3 discusses the probabilistic teleportation schemes using partially entangled GHZ state and two-qubit partially entangled state as quantum channel and the method for reducing classical communication cost. Another scheme is presented in Section 4 using another non-symmetric quantum channel combination (i.e., partially entangled W state and two-qubit partially entangled state). A method is given to combine unitary operations into one under the same basis as well. In Section 5 and Section 6, we present a discussion and conclude the whole paper.

## 2. System Model

In this paper, we consider two nodes, conveniently called Alice and Bob, who share entangled states as quantum channel. Through the channel, Alice wants to transmit arbitrary two-qubit state to Bob as described below

(1) $$|\chi \rangle =a_{0}|00\rangle +a_{1}|01\rangle +a_{2}|10\rangle +a_{3}|11\rangle ,$$

where $a_{i}\left(i=0,1,2,3\right)$ is the amplitude of respective basis state satisfying the normalized condition $\sum_{i=0}^{3}{\left|a_{i}\right|}^{2}=1$. The quantum channel shared between two nodes consists of a two-qubit partially entangled state and three-qubit partially entangled state. GHZ state and W state are fundamental entangled states of three qubits, and widely used in protocols for transmitting quantum states. They represent diverse types of but can cover all three-qubit entangled states. Without losing generality, both GHZ state and W state are studied as part of quantum channel combination but separately in different schemes. The two-qubit partially entangled state and three-qubit partially entangled states are described as

(2) $$\begin{array}{cc} |\psi \rangle =c|00\rangle +d|11\rangle , & \mathrm{where}\;{\left|c\right|}^{2}+{\left|d\right|}^{2}=1\;\mathrm{and}\;\left|c\right|\geq \left|d\right|,\\ |GHZ\rangle =m|000\rangle +n|111\rangle , & \mathrm{where}\;{\left|m\right|}^{2}+{\left|n\right|}^{2}=1\;\mathrm{and}\;\left|m\right|\geq \left|n\right|,\\ |W\rangle =x|001\rangle +y|010\rangle +z|100\rangle , & \mathrm{where}\;{\left|x\right|}^{2}+{\left|y\right|}^{2}+{\left|z\right|}^{2}=1\;\mathrm{and}\;\left|x\right|\geq \left|y\right|\geq \left|z\right|. \end{array}$$

**Figure 1:**
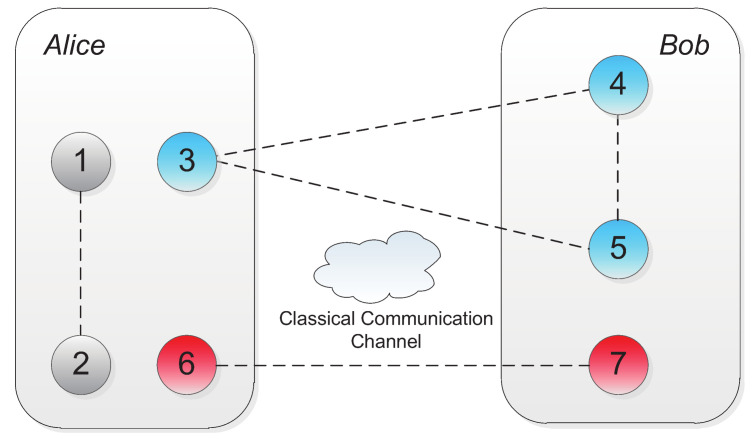
System model for teleporting arbitrary two-qubit state from Alice to Bob via non-symmetric quantum channel. For the convenience of description, we assume particles 1 and 2 are in the possession of Alice. Particle 3 from three-qubit partially entangled state is with Alice while Bob has particles 4 and 5. Particles 6 and 7 of two-qubit partially entangled state belong to Alice and Bob, respectively.

The system can be summarized into one general model as shown in Figure 1 where a two-qubit partially entangled state and a three-qubit partially entangled state are shared between Alice and Bob as a non-symmetric quantum channel. The classical communication channel is equipped.

In the following paper, the three-qubit partially entangled W state is firstly considered as quantum channel together with two-qubit partially entangled state. We refer to this scheme as scheme A and the channel combination as non-symmetric quantum channel combination A. In addition, a special case for teleporting two-qubit entangled state is discussed using the same quantum channel combination as scheme A. Then, the three-qubit partially entangled GHZ state is used to replace former partially entangled W state. Similarly, we refer to it as scheme B and the non-symmetric quantum channel combination B. In the following, we present these schemes in detail and give methods for improvement.

## 3. Schemes Using Non-Symmetric Quantum Channel Combination A

In this section, the scheme to transmit an arbitrary two-qubit state is presented when using defined non-symmetric quantum channel combination A. As a special case of scheme A, teleportation of two-qubit entangled state is discussed accompanied with a method for reducing the classical communication cost in that case.

### 3.1. Teleporting Arbitrary Two-Qubit Quantum State

The non-symmetric quantum channel utilized for probabilistic teleportation of arbitrary two-qubit state is composed of a two-qubit partially entangled state and three-qubit partially entangled W state. Taking the states described in Equation (2), the initial system state can be written as

(3) $$|\Phi_{sys}\rangle ={|\chi \rangle }_{12}⊗{|W\rangle }_{345}⊗{|\psi \rangle }_{67}.$$

To realize teleportation, the detailed process is elaborated as follows:

**Step 1**: Alice firstly performs two Bell-state measurements on particles (1, 3) and particles (2, 6), respectively. The system state may collapse into one of the 16 possible states, which can be expressed as ${\langle \beta_{ij}|}_{26}{\langle \beta_{kl}|}_{13}\Phi_{sys}\rangle$ in general, where $|\beta_{ij}\rangle$ and $|\beta_{kl}\rangle$ represent corresponding Bell states in the form

(4) $$|\beta_{ij}\rangle =\left(|0,j\rangle +{\left(-1\right)}^{i}|1,1-j\rangle \right)/\sqrt{2},$$

where i,j=0,1. Bell-state measurement results are expressed as classical bit strings $m_{p}m_{q}\left(|\beta_{ij}\rangle \right)\equiv ij,$ where $m_{p}m_{q}$ denote the measurement results of particle *p* and *q*, respectively. Then, Alice sends these measurement results to Bob through classical communication channel immediately. According to the measurement results $m_{1}m_{3}m_{2}m_{6}$, the 16 possible states can be divided into four groups as follows, where $\bar{m_{i}}$ indicates the negation of the measurement outcome $m_{i}$.

- When $m_{1}m_{3}m_{2}m_{6}$ is 0000, 0010, 1000 or 1010, i.e., $\bar{m_{3}}\;\bar{m_{6}}=1$, the system state is expressed as
  (5) $$\begin{array}{c} \frac{1}{2}(a_{0}xc|010\rangle +a_{0}yc|100\rangle +{\left(-1\right)}^{m_{1}}a_{2}zc|000\rangle +{\left(-1\right)}^{m_{2}}a_{1}xd|011\rangle \\ \;+{\left(-1\right)}^{m_{2}}a_{1}yd|101\rangle +{\left(-1\right)}^{m_{1}⊕m_{2}}a_{3}zd|001\rangle {)}_{457}. \end{array}$$
- When $m_{1}m_{3}m_{2}m_{6}$ is 0001, 0011, 1001 or 1011, i.e., $\bar{m_{3}}\;m_{6}=1$, the system state is expressed as
  (6) $$\begin{array}{c} \frac{1}{2}(a_{0}xd|011\rangle +a_{0}yd|101\rangle +{\left(-1\right)}^{m_{1}}a_{1}xc|010\rangle +{\left(-1\right)}^{m_{1}}a_{1}yc|100\rangle \\ \;+{\left(-1\right)}^{m_{2}}a_{2}zd|001\rangle +{\left(-1\right)}^{m_{1}⊕m_{2}}a_{3}zc|000\rangle {)}_{457}. \end{array}$$
- When $m_{1}m_{3}m_{2}m_{6}$ is 0100, 0110, 1100 or 1110, i.e., $m_{3}\;\bar{m_{6}}=1$, the system state is expressed as
  (7) $$\begin{array}{c} \frac{1}{2}(a_{0}zc|000\rangle +{\left(-1\right)}^{m_{2}}a_{1}zd|001\rangle +{\left(-1\right)}^{m_{1}}a_{2}xc|010\rangle +{\left(-1\right)}^{m_{1}}a_{2}yc|100\rangle \\ \;+{\left(-1\right)}^{m_{1}⊕m_{2}}a_{3}xd|011\rangle +{\left(-1\right)}^{m_{1}⊕m_{2}}a_{3}yd|101\rangle {)}_{457}. \end{array}$$
- When $m_{1}m_{3}m_{2}m_{6}$ is 0101, 0111, 1101 or 1111, i.e., $m_{3}\;m_{6}=1$, the system state is expressed as
  (8) $$\begin{array}{c} \frac{1}{2}(a_{0}zd|001\rangle +{\left(-1\right)}^{m_{1}}a_{2}xd|011\rangle +{\left(-1\right)}^{m_{1}}a_{2}yd|101\rangle +{\left(-1\right)}^{m_{2}}a_{1}zc|000\rangle \\ \;+{\left(-1\right)}^{m_{1}⊕m_{2}}a_{3}xc|010\rangle +{\left(-1\right)}^{m_{1}⊕m_{2}}a_{3}yc|100\rangle {)}_{457}. \end{array}$$

**Step 2**: After receiving the classical information from Alice, Bob performs projective measurement on particle 4. If the result is ${|0\rangle }_{4}$ (denoted by $m_{4}=0$), the original state can not be reconstructed and the teleportation fails. Otherwise, Bob continues to apply following operations to recover the teleported quantum state.

**Step 3**: Then, for retrieving the correspondence between coefficients $a_{i}$ and basis states, Bob needs to apply unitary operation $U_{57}$ on particles (5, 7). The specific unitary operation required is determined by the measurement result according to the formula

(9) $$U_{57}={\left(Z^{m_{1}}X^{\bar{m_{3}}}\right)}_{5}⊗{\left(Z^{m_{2}}X^{m_{6}}\right)}_{7},$$

where X=0110 and Z=100−1 are *Pauli* matrices. After unitary operation $U_{57}$, the specific system state is determined by $m_{3}m_{6}$ and changes into

(10) $$|\Phi_{sys}^{\prime }\rangle =\left\{\begin{array}{cc} a_{0}xc|00\rangle +a_{1}xd|01\rangle +a_{2}zc|10\rangle +a_{3}zd|11\rangle & \mathrm{when}\;\bar{m_{3}}\;\bar{m_{6}}=1,\\ a_{0}xd|00\rangle +a_{1}xc|01\rangle +a_{2}zd|10\rangle +a_{3}zc|11\rangle & \mathrm{when}\;\bar{m_{3}}\;m_{6}=1,\\ a_{0}zc|00\rangle +a_{1}zd|01\rangle +a_{2}xc|10\rangle +a_{3}xd|11\rangle & \mathrm{when}\;m_{3}\;\bar{m_{6}}=1,\\ a_{0}zd|00\rangle +a_{1}zc|01\rangle +a_{2}xd|10\rangle +a_{3}xc|11\rangle & \mathrm{when}\;m_{3}\;m_{6}=1. \end{array}\right.$$

**Step 4**: Bob introduces an auxiliary particle A with its initial state ${|0\rangle }_{A}$ and applies a collective unitary operation on particles (5, 7, A). To reconstruct the original state under the basis $\left\{{|\beta_{ij}\rangle }_{57}{|0\rangle }_{A},{|\beta_{ij}\rangle }_{57}{|1\rangle }_{A}\right\}$ (where ${|\beta_{ij}\rangle }_{57}$ stands for the computational basis of an four-dimensional Hilbert space), the unitary operation should take the form

(11) $$\begin{array}{c} U_{57A}=\left(\begin{array}{cc} C_{1} & C_{2}\\ C_{2} & -C_{1} \end{array}\right). \end{array}$$

The $C_{i}\left(i=1,2\right)$ are 4×4 matrices in the form

(12) $$\left\{\begin{array}{c} C_{1}=diag\left(c_{1},c_{2},c_{3},c_{4}\right),\\ C_{2}=diag\left(\sqrt{1-{c_{1}}^{2}},\sqrt{1-{c_{2}}^{2}},\sqrt{1-{c_{3}}^{2}},\sqrt{1-{c_{4}}^{2}}\right), \end{array}\right.$$

where $c_{i}$ (i=1,2,3,4 and $\left|c_{i}\right|\leq 1$) and their corresponding $C_{i}$ all depend on the specific system state. The specific form of $c_{i}$ is summarized into the following expressions:

(13) $$C_{1}=\left\{\begin{array}{cc} diag(\frac{zd}{xc},\frac{z}{x},\frac{d}{c},1) & \mathrm{when}\;\bar{m_{3}}\;\bar{m_{6}}=1,\\ diag(\frac{z}{x},\frac{zd}{xc},1,\frac{d}{c}) & \mathrm{when}\;\bar{m_{3}}\;m_{6}=1,\\ diag(\frac{d}{c},1,\frac{zd}{xc},\frac{z}{x}) & \mathrm{when}\;m_{3}\;\bar{m_{6}}=1,\\ diag(1,\frac{d}{c},\frac{z}{x},\frac{zd}{xc}) & \mathrm{when}\;m_{3}\;m_{6}=1. \end{array}\right.$$

**Step 5**: Finally, Bob performs projective measurement on particle A. The result ${|1\rangle }_{A}$ (denoted by $m_{A}=1$) indicates the failure of this teleportation; on the contrary, if the result is $m_{A}=0$, the two-qubit state has been reconstructed on particles 5 and 7, yielding a successful teleportation.

**Figure 2:**
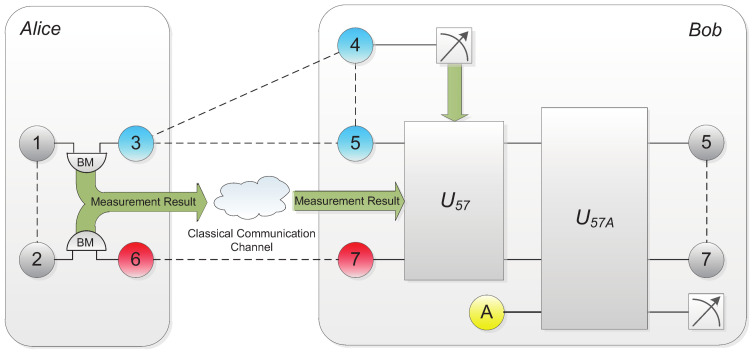
Probabilistic teleportation scheme utilizing partially entangled two-qubit state and W state as quantum channel. Bell-state measurements, projective measurements and local unitary operations are applied, together with the classical information, for original state recovery.

The success probability of scheme A is $4{\left|zd\right|}^{2}$. When $\left|x\right|=\left|y\right|=\left|z\right|=1/\sqrt{3}$ and $\left|c\right|=\left|d\right|=1/\sqrt{2}$, i.e., the quantum channel consists of two maximally entangled states, the success probability would reach its maximum 2/3. The whole scheme is shown in Figure 2 and an example is given for illustrating the whole process better.

**Example** **1.** *Assume the Bell-state measurement results $m_{1}m_{3}m_{2}m_{6}=0000$. According to Equation *(5)*, the system state after Alice’s two Bell-state measurements should be*

$${\langle \beta_{00}|}_{26}{\langle \beta_{00}|}_{13}\Phi_{sys}\rangle =\frac{1}{2}{\left(a_{0}xc|010\rangle +a_{0}yc|100\rangle +a_{2}zc|000\rangle +a_{1}xd|011\rangle +yd|101\rangle +a_{3}zd|001\rangle \right)}_{457}.$$

*Bob then measures particle 4. If the result is $m_{4}=1$, the teleportation fails. Otherwise, he continues to apply unitary operation $U_{57}=X_{5}⊗I_{7}$ on particles (5, 7) according to Equation *(9)*. After that, Bob introduces an auxiliary particle A with its initial state ${|0\rangle }_{A}$ and the system state changes into*

$$|\Phi_{sys}^{\prime \prime }\rangle =\frac{1}{2}{\left(a_{0}xc|000\rangle +a_{1}xd|010\rangle +a_{2}zc|100\rangle +a_{3}zd|110\rangle \right)}_{57A}.$$

*Choosing $C_{1}=diag\left(\frac{zd}{xc},\frac{z}{x},\frac{d}{c},1\right)$, corresponding eight-dimensional unitary operation $U_{57A}$ is made up as below according to Equations *(11)* and *(12)*.*

$$U_{57A}=\left[\begin{array}{cccccccc} \frac{zd}{xc} & 0 & 0 & 0 & \sqrt{1-{\left(\frac{zd}{xc}\right)}^{2}} & 0 & 0 & 0\\ 0 & \frac{z}{x} & 0 & 0 & 0 & \sqrt{1-{\left(\frac{z}{x}\right)}^{2}} & 0 & 0\\ 0 & 0 & \frac{d}{c} & 0 & 0 & 0 & \sqrt{1-{\left(\frac{d}{c}\right)}^{2}} & 0\\ 0 & 0 & 0 & 1 & 0 & 0 & 0 & 0\\ \sqrt{1-{\left(\frac{zd}{xc}\right)}^{2}} & 0 & 0 & 0 & -\frac{zd}{xc} & 0 & 0 & 0\\ 0 & \sqrt{1-{\left(\frac{z}{x}\right)}^{2}} & 0 & 0 & 0 & -\frac{z}{x} & 0 & 0\\ 0 & 0 & \sqrt{1-{\left(\frac{d}{c}\right)}^{2}} & 0 & 0 & 0 & -\frac{d}{c} & 0\\ 0 & 0 & 0 & 0 & 0 & 0 & 0 & -1 \end{array}\right].$$

*After the operation, Bob obtains the system state*

$$\begin{array}{cc} |\Phi_{final}\rangle =U_{57A}|\Phi_{sys}^{\prime \prime }\rangle = & \frac{zd}{2}\left(a_{0}{|00\rangle }_{57}+a_{1}{|01\rangle }_{57}+a_{2}{|10\rangle }_{57}+a_{3}{|11\rangle }_{57}\right){|0\rangle }_{A}\\ & +\left(\sqrt{x^{2}c^{2}-z^{2}d^{2}}a_{0}{|00\rangle }_{57}+d\sqrt{x^{2}-z^{2}}a_{1}{|01\rangle }_{57}+z\sqrt{c^{2}-d^{2}}a_{2}{|10\rangle }_{57}\right){|1\rangle }_{A}. \end{array}$$

*Then, undertaking measurements on particle A by Bob, the result $m_{A}=0$ indicates that the original state has been recovered at Bob (on particles 5 and 7) successfully while $m_{A}=1$ means failure of teleportation. Using a two-qubit partially entangled state and a three-qubit partially entangled W state as quantum channel, we could teleport arbitrary two-qubit state probabilistically.*

### 3.2. Teleporting Two-Qubit Entangled State

In this subsection, we apply scheme A on teleportation of two-qubit entangled state. In addition, we introduce a new method of processing classical information that will help to reduce the classical communication cost needed in this case. Without losing generality, the two-qubit entangled state to be teleported is assumed in the general form $|\gamma \rangle =\alpha |00\rangle +\beta |11\rangle ,$ where ${\left|\alpha \right|}^{2}+{\left|\beta \right|}^{2}=1$ and $\left|\alpha \right|\geq \left|\beta \right|>0$. This form is widely used in related works and any entangled two-qubit state can be brought to this form via local unitary operations. Alice still performs Bell-state measurements on particles (1, 3) and particles (2, 6), respectively. The system state would collapse into one of the 16 possible states, which are classified according to measurement results as follows:

(14) $$|\Phi_{sys}^{\prime }\rangle =\left\{\begin{array}{cc} \frac{1}{2}{\left(\alpha xc|010\rangle +\alpha yc|100\rangle +{\left(-1\right)}^{m_{1}⊕m_{2}}\beta zd|001\rangle \right)}_{457} & \mathrm{when}\;\bar{m_{3}}\;\bar{m_{6}}=1,\\ \frac{1}{2}{\left(\alpha xd|011\rangle +\alpha yd|101\rangle +{\left(-1\right)}^{m_{1}⊕m_{2}}\beta zc|000\rangle \right)}_{457} & \mathrm{when}\;\bar{m_{3}}\;m_{6}=1,\\ \frac{1}{2}{\left(\alpha zc|000\rangle +{\left(-1\right)}^{m_{1}⊕m_{2}}\beta xd|011\rangle +{\left(-1\right)}^{m_{1}⊕m_{2}}\beta yd|101\rangle \right)}_{457} & \mathrm{when}\;m_{3}\;\bar{m_{6}}=1,\\ \frac{1}{2}{\left(\alpha zd|001\rangle +{\left(-1\right)}^{m_{1}⊕m_{2}}\beta xc|010\rangle +{\left(-1\right)}^{m_{1}⊕m_{2}}\beta yc|100\rangle \right)}_{457} & \mathrm{when}\;m_{3}\;m_{6}=1. \end{array}\right.$$

The projective measurement on particle 4 is also performed by Bob after receiving the classical information from Alice. When measurement result of particle 4 is $m_{4}=0$, Bob continues to apply unitary operation $U_{57}$ on particles (5, 7) as

(15) $$U_{57}={\left(X^{\bar{m_{3}}}\right)}_{5}⊗{\left(Z^{m_{1}⊕m_{2}}X^{m_{6}}\right)}_{7}.$$

An auxiliary particle A is introduced with its initial state ${|0\rangle }_{A}$, and then the collective unitary operation $U_{57A}$ is constructed by Bob. The $U_{57A}$ takes the same form with Equations (11)–(13) because the unitary operation is only related with the quantum channel characters. Measurement on particle A is still required so that Bob can judge whether this teleportation succeeds or not. The total success probability of teleportation is $4{\left|zd\right|}^{2}$.

Comparing these two schemes, the main difference is the formulation of $U_{57}$, which is closely related with the Bell-state measurement results sent from Alice. The original four cbits information $m_{1}m_{3}m_{2}m_{6}$ is sent to Bob directly through classical communication channel. However, after observing Equation (15), we get the conclusion that whether the Z operation on particle 7 is required or not is determined by the *XOR* result between $m_{1}$ and $m_{2}$. Thus, we use $m_{x}=m_{1}⊕m_{2}$ to denote the XOR result and the Equation (15) can be rewritten as

(16) $$U_{57}={\left(X^{\bar{m_{3}}}\right)}_{5}⊗{\left(Z^{m_{x}}X^{m_{6}}\right)}_{7}.$$

Through this combination, only the *XOR* result $m_{x}$ instead of the respective measurement results of particles 1 and 2 needs to be sent to Bob together with $m_{3}m_{6}$. These 3 cbits information rather than 4 cbits are enough for Bob to determine $U_{57}$ so that the classical communication cost is reduced by 25%, which is one of the advantages of this scheme.

**Remark** **1.** Actually, if setting the parameter $a_{1}=a_{2}=0$ in Equation *(1)*, we can also get the case discussed above. Scheme A for teleporting arbitrary two-qubit state is a more general one where the scheme for teleporting two-qubit entangled state can be seen as a special case. The method presented for reducing the classical communication cost is only valid in this case. If the two-qubit entangled state is given in other forms, the expression of $U_{57}$ would change accordingly. However, for avoiding re-derivation, Alice could use the result-mapping method proposed in [25] to obtain the operation $U_{57}$ correctly.

## 4. Scheme Using Non-Symmetric Quantum Channel Combination B

In the schemes above, the maximal success probability of teleportation could only reach 2/3 even if the quantum channel is composed of corresponding maximally entangled states. Analyzing the schemes, the measurement on particle 4 is the main source of the non-fully recoverable system state. In this section, the three-qubit partially entangled GHZ state is utilized in quantum channel combination B and the corresponding scheme using such quantum channel to complete arbitrary two-qubit state teleportation is presented. The initial system state is expressed as

(17) $$|\Phi_{sys}\rangle ={|\chi \rangle }_{12}⊗{|GHZ\rangle }_{345}⊗{|\psi \rangle }_{67},$$

where ${|GHZ\rangle }_{345}$ is partially entangled GHZ state with the form as Equation (2). Similar to aforementioned schemes, Alice performs two Bell-state measurements on particles (1, 3) and particles (2, 6), respectively. The system state collapses and can be divided into four groups as follows:

- When $m_{1}m_{3}m_{2}m_{6}$ is 0000, 0010, 1000 or 1010, i.e., $\bar{m_{3}}\;\bar{m_{6}}=1$, the system state is expressed as
  (18) $$\frac{1}{2}{\left(a_{0}mc|000\rangle +{\left(-1\right)}^{m_{2}}a_{1}md|001\rangle +{\left(-1\right)}^{m_{1}}a_{2}nc|110\rangle +{\left(-1\right)}^{m_{1}⊕m_{2}}a_{3}nd|111\rangle \right)}_{457}.$$
- When $m_{1}m_{3}m_{2}m_{6}$ is 0001, 0011, 1001 or 1011, i.e., $\bar{m_{3}}\;m_{6}=1$, the system state is expressed as
  (19) $$\frac{1}{2}{\left(a_{0}md|001\rangle +{\left(-1\right)}^{m_{2}}a_{1}mc|000\rangle +{\left(-1\right)}^{m_{1}}a_{2}nd|111\rangle +{\left(-1\right)}^{m_{1}⊕m_{2}}a_{3}nc|110\rangle \right)}_{457}.$$
- When $m_{1}m_{3}m_{2}m_{6}$ is 0100, 0110, 1100 or 1110, i.e., $m_{3}\;\bar{m_{6}}=1$, the system state is expressed as
  (20) $$\frac{1}{2}{\left(a_{0}nc|110\rangle +{\left(-1\right)}^{m_{2}}a_{1}nd|111\rangle +{\left(-1\right)}^{m_{1}}a_{2}mc|000\rangle +{\left(-1\right)}^{m_{1}⊕m_{2}}a_{3}md|001\rangle \right)}_{457}.$$
- When $m_{1}m_{3}m_{2}m_{6}$ is 0101, 0111, 1101 or 1111, i.e., $m_{3}\;m_{6}=1$, the system state is expressed as
  (21) $$\frac{1}{2}{\left(a_{0}nd|111\rangle +{\left(-1\right)}^{m_{2}}a_{1}nc|110\rangle +{\left(-1\right)}^{m_{1}}a_{2}md|001\rangle +{\left(-1\right)}^{m_{1}⊕m_{2}}a_{3}mc|000\rangle \right)}_{457}.$$

**Figure 3:**
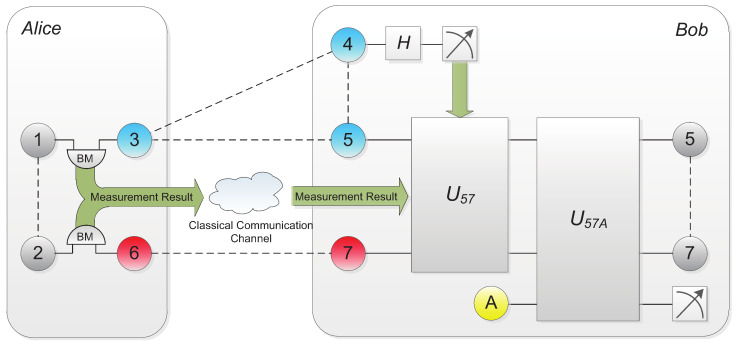
Probabilistic teleportation scheme utilizing partially entangled two-qubit state and GHZ state as quantum channel. Extra H operation is applied before operation $U_{57}$ to avoid failure of teleportation.

The main difference of this scheme is that Bob needs to send particle 4 through additional *Hadamard* gate (H=12111−1) before measurement as shown in Figure 3. Afterwards, Bob performs projective measurement on particle 4. The unitary operation $U_{57}$ performed on particles (5, 7) to retrieve the correspondence would be determined by the measurement result $m_{4}$ together with $m_{1}m_{3}m_{2}m_{6}$ through the following formula:

(22) $$U_{57}={\left(-1\right)}^{m_{3}m_{4}}{\left(Z^{m_{1}⊕m_{4}}X^{m_{3}}\right)}_{5}⊗{\left(Z^{m_{2}}X^{m_{6}}\right)}_{7}.$$

The system state changes into

(23) $$|\Phi_{sys}^{\prime }\rangle =\left\{\begin{array}{cc} \frac{1}{2\sqrt{2}}{\left(a_{0}mc|00\rangle +a_{1}md|01\rangle +a_{2}nc|10\rangle +a_{3}nd|11\rangle \right)}_{57} & \mathrm{when}\;\bar{m_{3}}\;\bar{m_{6}}=1,\\ \frac{1}{2\sqrt{2}}{\left(a_{0}md|00\rangle +a_{1}mc|01\rangle +a_{2}nd|10\rangle +a_{3}nc|11\rangle \right)}_{57} & \mathrm{when}\;\bar{m_{3}}\;m_{6}=1,\\ \frac{1}{2\sqrt{2}}{\left(a_{0}nc|00\rangle +a_{1}nd|01\rangle +a_{2}mc|10\rangle +a_{3}md|11\rangle \right)}_{57} & \mathrm{when}\;m_{3}\;\bar{m_{6}}=1,\\ \frac{1}{2\sqrt{2}}{\left(a_{0}nd|00\rangle +a_{1}nc|01\rangle +a_{2}md|10\rangle +a_{3}mc|11\rangle \right)}_{57} & \mathrm{when}\;m_{3}\;m_{6}=1. \end{array}\right.$$

Then, Bob introduces an auxiliary particle A and performs a collective unitary operation $U_{57A}$ on particles (5, 7, A) to correct the distortion on system state and recover the original state. The transformation matrix of $U_{57A}$ should be constructed in accordance with Equations (11) and (12). The exact form of $C_{1}$ is also determined by $m_{3}m_{6}$ and expressed as

(24) $$C_{1}=\left\{\begin{array}{cc} diag(\frac{nd}{mc},\frac{n}{m},\frac{d}{c},1) & \mathrm{when}\;\bar{m_{3}}\;\bar{m_{6}}=1,\\ diag(\frac{n}{m},\frac{nd}{mc},1,\frac{d}{c}) & \mathrm{when}\;\bar{m_{3}}\;m_{6}=1,\\ diag(\frac{d}{c},1,\frac{nd}{mc},\frac{n}{m}) & \mathrm{when}\;m_{3}\;\bar{m_{6}}=1,\\ diag(1,\frac{d}{c},\frac{n}{m},\frac{nd}{mc}) & \mathrm{when}\;m_{3}\;m_{6}=1. \end{array}\right.$$

After operation $U_{57A}$, projective measurement is performed on particle A. Similarly, only the result $m_{A}=0$ indicates successful teleportation. The total success probability is $4{\left|nd\right|}^{2}$. If $\left|m\right|=\left|n\right|=\left|c\right|=\left|d\right|=1/\sqrt{2}$, i.e., the quantum channel is composed of two maximally entangled states, the success probability would reach its maximum of 1. With the help of partially entangled GHZ state and additional H operation, scheme B could increase the maximal success probability by avoiding failure occurring after measurement on particle 4.

In the above schemes, Bob applies two separate unitary operations under different bases. $U_{57}$ is constructed under computational basis while $U_{57A}$ is made under the basis of $\left\{{|\beta_{ij}\rangle }_{57}{|0\rangle }_{A},{|\beta_{ij}\rangle }_{57}{|1\rangle }_{A}\right\}$. These two unitary operations can not be combined directly through tensor product or matrix multiplication. For combining them into one operation under unified basis, we introduce the transformation matrix *T* between these two bases. With this matrix, $U_{57}$ and $U_{57A}$ could be combined into one complete unitary operation under computational basis. The detailed method is shown in the following part and the basis transformation matrix *T* is given as

(25) $$T=\left[\begin{array}{cccccccc} 1 & 0 & 0 & 0 & 0 & 0 & 0 & 0\\ 0 & 0 & 1 & 0 & 0 & 0 & 0 & 0\\ 0 & 0 & 0 & 0 & 1 & 0 & 0 & 0\\ 0 & 0 & 0 & 0 & 0 & 0 & 1 & 0\\ 0 & 1 & 0 & 0 & 0 & 0 & 0 & 0\\ 0 & 0 & 0 & 1 & 0 & 0 & 0 & 0\\ 0 & 0 & 0 & 0 & 0 & 1 & 0 & 0\\ 0 & 0 & 0 & 0 & 0 & 0 & 0 & 1 \end{array}\right].$$

Based on the existing unitary operation $U_{57A}$ in Equation (22), the new unitary operation ${\hat{U}}_{57A}$ under computational basis can be obtained through ${\hat{U}}_{57A}=T^{-1}U_{57A}T$, where the matrix *T* is equivalent to transition matrix actually. Hence, Bob could introduce the auxiliary particle ${|0\rangle }_{A}$ after obtaining the measurement result of particle 4 but before performing unitary operation. With the newly constructed unitary operation matrix ${\hat{U}}_{57A}$, the two unitary operations can be combined into one operation $U^{\prime }$ under computational basis easily in the form of

(26) $$U^{\prime }={\hat{U}}_{57A}\left(U_{57}⊗I_{A}\right),$$

where $I_{A}$ is a two-dimensional identity matrix. We illustrate this method by taking $m_{1}m_{3}m_{2}m_{6}m_{4}=01110$ as an example. According to Equation (21), after measurement on particle 4 and introducing auxiliary particle ${|0\rangle }_{A}$, the system state should be

(27) $$|\Phi_{sys}^{\prime \prime }\rangle =\frac{1}{2\sqrt{2}}{\left(-a_{3}mc|000\rangle +a_{2}md|010\rangle -a_{1}nc|100\rangle +a_{0}nd|110\rangle \right)}_{57A},$$

which can be expressed with vector under computational basis as

$$|\Phi_{sys}^{\prime \prime }\rangle =\frac{1}{2\sqrt{2}}{\left[-a_{3}mc,0,a_{2}md,0,-a_{1}nc,0,a_{0}nd,0\right]}^{T}.$$

Then, perform the combined unitary operation $U^{\prime }$

(28) $$U^{\prime }={\hat{U}}_{57A}\left(U_{57}⊗I_{A}\right)=T^{-1}\left(\begin{array}{cc} C_{1} & C_{2}\\ C_{2} & -C_{1} \end{array}\right)T\left(X_{5}⊗{\left(ZX\right)}_{7}⊗I_{A}\right),$$

where $C_{1}=diag\left(1,\frac{d}{c},\frac{n}{m},\frac{nd}{mc}\right)$ accordingly so that the final system state is

(29) $$\begin{array}{cc} |\Phi_{final}\rangle & =U^{\prime }|\Phi_{sys}^{\prime \prime }\rangle =\frac{1}{2\sqrt{2}}{\left[a_{0}nd,0,a_{1}nd,a_{1}n\sqrt{c^{2}-d^{2}},a_{2}nd,a_{2}d\sqrt{m^{2}-n^{2}},a_{3}nd,a_{3}\sqrt{c^{2}m^{2}-d^{2}n^{2}}\right]}^{T}\\ & =\frac{dn}{2\sqrt{2}}{\left(a_{0}|00\rangle +a_{1}|01\rangle +a_{2}|10\rangle +a_{3}|11\rangle \right)}_{57}{|0\rangle }_{A}\\ & \;+\frac{1}{2\sqrt{2}}{\left(a_{1}n\sqrt{c^{2}-d^{2}}|01\rangle +a_{2}d\sqrt{m^{2}-n^{2}}|10\rangle +a_{3}\sqrt{c^{2}m^{2}-d^{2}n^{2}}|11\rangle \right)}_{57}{|1\rangle }_{A}. \end{array}$$

Similarly, we need measurement on auxiliary particle A. The measurement result $m_{A}=0$ indicates successful teleportation. On the contrary, $m_{A}=1$ suggests failure.

**Remark** **2.** Obviously, only one unitary operation is performed instead of the previous two separate operations, which would simplify the quantum manipulation. Less operation may lead to reduction in the possibility of making an error in practice. This method can also be applied in our previous presented schemes compatibly, and the whole teleportation process may benefit from simplified operation. In addition, when the quantum channel is composed of maximally entangled states, the success probability may reach its maximum. In that situation, the system state after operation $U_{57}$ had already been recovered to its original state successfully. There is no need to introduce auxiliary particle and apply operation $U_{57A}$ any more.

## 5. Discussion

We present schemes utilizing two different non-symmetric quantum channel combinations to teleport arbitrary two-qubit state probabilistically in this paper. One scheme consists of two-qubit partially entangled state and three-qubit partially entangled W state, and the other one consists of two-qubit partially entangled state and three-qubit partially entangled GHZ state. We still refer to these two schemes as scheme A and scheme B in the following discussion and conclusion.

(1) The belonging of particle 4

In the paper, we assume that particle 4 from the three-qubit partially entangled state is held by Bob. However, the belonging of the particle should be flexible. There are still two different situations in addition to what we have considered. We will analyze them case by case to show that our schemes are also applicable in the situations where Bob does not have particle 4. (a) When Alice has particle 4, Bob only has particles 5 and 7. In this situation, Alice should perform the measurement on particle 4 after the Bell-state measurements in scheme A or after H operation in scheme B, and then send Bob the result. If Alice gets the result $m_{4}=1$ in scheme A, she should stop the whole teleportation process and restart another one immediately. Otherwise, if the result is $m_{4}=0$, Alice should send measurement results to Bob, and then Bob continues to apply the corresponding operation for recovering the original state. (b) When particle 4 belongs to a third party Charles, the system changes to a controlled teleportation model where the measurement on particle 4 should be performed by Charles. As a trusted third party, Charles can control the whole teleportation because Bob cannot recover the original state and complete the teleportation without Charles’ cooperation (measurement and its result). In a real application, the reasonable allocation of particle 4 should be determined according to specific condition and purpose. Our presented schemes can work well in all three of these three situations with few modifications.

(2) Another understanding of our schemes

In both schemes, Alice needs to perform two Bell-state measurements on particles firstly and send measurement results to Bob through classical communication channels. Then, Bob performs the measurement on particle 4. If a partially entangled GHZ state is utilized, an extra H operation should be applied before Bob’s measurement. After that, an auxiliary particle is introduced for reconstructing original state by applying specific unitary operation(s) according to their measurement results.

If analyzed from another point of view, the measurement on particle 4 of three-qubit partially entangled W state in scheme A and operations (H operation and measurement) on particle 4 of three-qubit partially entangled GHZ state can be seen as the process of preparing a two-qubit entangled state for the teleportation protocol. The remainder of the teleportation can then be analyzed using previously explored techniques [26]. We will refer to this as Scheme R for the discussion below.

This intuitive understanding of the protocol may help us to explain why maximum success probability of scheme A using W state can not reach 1. This is primarily because a two-qubit partially entangled state can only be obtained with some probability from the remainder of the two-qubit system of the W state after measurement. The probability is exactly 2/3, which limits the maximum of success probability of the whole teleportation. The result of measurement on particle 4 indicates whether the two-qubit entangled channel is prepared successfully. In contrast, we can obtain a two-qubit partially entangled state with certainty from the operations on particle 4 of the partially entangled GHZ state so that the maximum success probability of scheme B can reach 1.

Comparing scheme R with our schemes, the main difference is the order of performing measurement on particle 4 while other manipulations are similar [26]. In scheme R, when Alice intends to teleport the two-qubit state, she needs to notify Bob to perform measurement on particle 4 to initiate the whole teleportation process. Then, Bob sends back information to Alice with notification that the channel is ready so that Alice can perform the Bell measurement and follow-up steps using the prepared two two-qubit entangled states as the channel. Obviously, there are two additional classical communication processes compared with our schemes. Extra classical communication cost for protocol control and more transmission delay will be introduced especially in the system model discussed in this paper.

However, if Alice holds particle 4 of the three-qubit partially entangled state, the additional communication processes of scheme R are unnecessary. Alice does not need to ask Bob to perform the measurement: instead she could do the preparation herself and be aware of whether the channel is ready. In addition, Alice could terminate the follow-up steps and restart another teleportation if she failed to get the two-qubit partially entangled state from the W state (when the result is 1), which would increase the whole success probability.

Based on the above analysis, our schemes proposed in paper may avoid introducing extra cost for the whole teleportation process. They are still meaningful and can be applied in specific scenes when necessary. This provides one feasible choice of teleportation scheme for two-qubit state transmitting.

## 6. Conclusions

In future quantum networks, the quantum states to be teleported and the entangled states shared among nodes are diverse. We studied probabilistic teleportation of two-qubit quantum states using partially entangled states as a channel in this paper. Two schemes were presented using non-symmetric quantum channels, which is different from the existing work. The quantum channel consists of a two-qubit partially entangled state and a three-qubit partially entangled state. Both GHZ state and W state were considered as representatives of three-qubit entangled states to give more complete solutions. The composite quantum channel we discussed may exist in real applications. To some extent, our schemes are supplementary to the protocol family of two-qubit state teleportation.

In addition, we illustrated the whole teleportation process in detail and the unitary operations required were given in concise formulas rather than tables. The required operation can be worked out through calculation instead of searching through complex tables, which is helpful in fast automatic control and processing. A method for reducing the classical communication cost in the special case of teleporting two-qubit entangled state was provided. By sending only 3 cbits compressed Bell-state measurement outcome to the receiver instead of 4 cbits, as in other related works, the cost could be reduced by 25%. Finally, the transformation matrix *T* was provided for converting unitary operation under different bases used. With matrix *T*, the former two separate unitary operations under different bases can be combined into one under unified computational basis. Less operation is associated with simpler manipulation and reduced possibility of error in theory. Furthermore, our schemes are applicable in other situations where there is some flexibility regarding where the particle belongs, as we discussed.

Schemes using partially entangled states to realize probabilistic teleportation are crucial for the practical application of quantum communication and networks. We hope our work may stimulate more investigations into proposals for quantum communication and networks. In future work, we plan to study the teleportation in the presence of unavoidable noises and test the efficiency of the protocol.
